# Dataset of close-range soil images and corresponding particle size distributions

**DOI:** 10.1016/j.dib.2025.111631

**Published:** 2025-05-12

**Authors:** E. Soranzo

**Affiliations:** Department of Civil Engineering and Natural Hazards, Peter Jordan Straße 82, Vienna, 1190, Austria

**Keywords:** Geotechnical engineering, Image analysis, Machine learning, Particle size distribution, Soil classification, Soil images

## Abstract

This dataset comprises close-range soil images and their corresponding particle size distributions, which are essential for various geotechnical applications. The images were captured with two different smartphones under standardized laboratory conditions using a dark chamber with constant lighting to ensure consistency. The dataset includes a diverse range of soil samples, from clayey silt to gravel. Each image is paired with particle size distribution data. This comprehensive dataset supports the development and validation of machine learning models for soil analysis and can be used for further research in geotechnical engineering, such as soil particle size prediction models.

## Highlights

• Dataset includes close-range soil images and particle size distributions.
• Diverse homogenized and oven-dried soil samples from clayey silt to gravel are included.
• Images taken with two different smartphone cameras in a dark chamber to minimize light reflection.
• Dataset was used to train a convolutional neural network (CNN) model for predicting particle size distributions [15].

Specification Table

**Table:** 

Subject	Geotechnical Engineering
Specific subject area	Soil Characterization
Type of data	Images and CSV file
Data collection	Dark chamber
Data-source location	Austria
Data accessibility	[1]
Related research articles	[2]

## 1 Value of the data

• The dataset comprises 136 high-resolution images and 26 CSV files, providing a rich source of data for soil particle size distribution analysis. This extensive collection allows for detailed examination and comparison of various soil samples, enhancing the reliability of the analysis.
• Images were taken using two different smartphones under controlled conditions in a dark chamber, ensuring minimal light reflection and consistent image quality. This standardization helps in reducing variability and improving the accuracy of subsequent analyses.
• The dataset includes a wide range of soil samples, from fine clayey silt to coarse gravel, collected from various locations. This diversity enhances the robustness and generalizability of the dataset, making it suitable for training and validating machine learning models aimed at predicting soil characteristics.
• Detailed particle soil distribution data, obtained through standardized sieving and sedimentation methods, accompany the images, offering comprehensive insights into soil properties.
• This dataset is ideal for training and validating machine learning models aimed at predicting soil characteristics, supporting advancements in geotechnical engineering.
• By providing high-quality, annotated soil images and particle size distribution data, the dataset facilitates further research and practical applications in soil mechanics and related fields.

## 2 Background

The dataset was collected at the Laboratory of Geotechnical Engineering of BOKU University for various clients, including building companies, state-owned enterprises, federal states and municipalities. The primary motivation for compiling this dataset was to obtain the particle size distribution (PSD) of soils, a critical factor in geotechnical engineering that affects various soil properties.

The resulting data provide information about the soil’s grain size distribution, which is crucial for understanding soil properties, including hydraulic conductivity [3], [4] and soil friction angle [5], [6], among others. Some correlations exist between the effective grain diameter $D_{10}$ and the hydraulic conductivity, as well as $D_{10}$, the coefficient of uniformity $C_{U}=D_{60}/D_{10}$ and soil friction angle, where $D_{10}$ and $D_{60}$ are the particle sizes such that 10 % and 60 % of the particles by weight are smaller than those sizes [7]. Information about the hydraulic conductivity and friction angle is essential for the design and analysis of foundations, earthworks and other geotechnical structures.

## 3 Data description

**Fig. 1:**
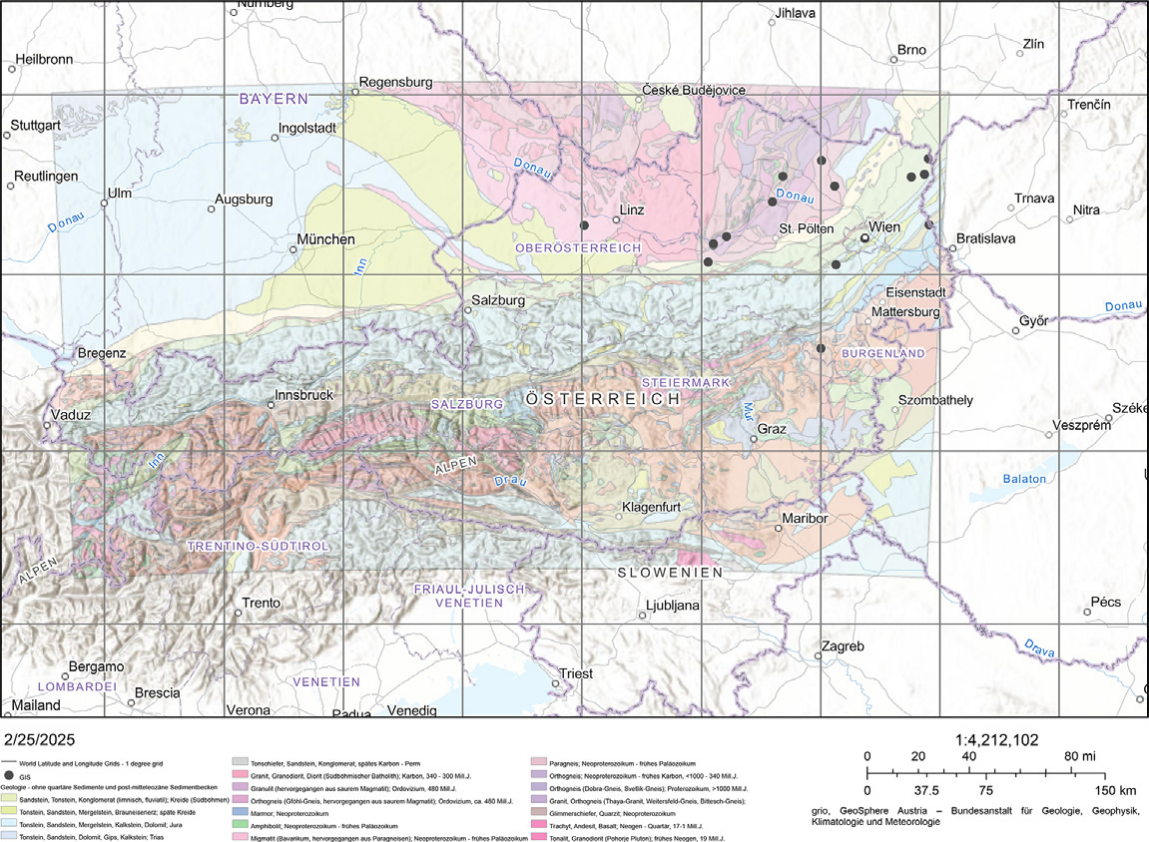
GIS visualization of the locations where soil samples were collected (indicated by black dots). Additionally, one sample was collected from Nepal [2].

There are 136 JPG images and 26 CSV files in the dataset. Images’ naming follows this structure: Smartphone_Sample_ID.jpg. Photos are taken with two smartphones (Smartphone) on the same soil samples (Sample). More than one photo (ID) is taken for each sample. Between the photographs, the soil surface is modified using various tools. For this reason, there are more images (136) than particle soil distributions (26). Table 1 summarizes the key data characteristics, grouping the soils per sample. The soil locations are depicted in Fig. 1. The CSV files are listed under Sample.csv and comprise the following columns:

• diam Particle diameter in mm
• finer Percentage of finer mass in %

**Table 1:** Summary of key data characteristics by location.

Sample	Location	Classifications													
		Geotechnical [8]										USDA [9]			
		Fractions		$d_{50}$	$C_{U}$	$C_{C}$	Co	Gr	Sa	Si	Cl	Texture class	Sa	Si	Cl
		Main	Secondary	(mm)	(%)	(%)	(%)	(%)	(%)	(%)	(%)		(%)	(%)	(%)
F827	Zistersdorf, AT	Si	sa	0.021	8.5	1.3	0.0	0.0	10.4	80.1	9.5	Silt loam	18.7	75.1	6.2
G190	Aichdorf bei Judenburg, AT	Si	sa	0.073	27.5	4.6	0.0	9.0	40.7	45.1	5.2	Sandy loam	58.0	35.4	6.4
H030	Wien, AT	Sa	si	0.100	18.8	3.0	0.0	2.0	60.7	30.5	6.8	Sandy loam	65.9	30.2	3.8
H031	St. Corona am Wechsel, AT	Gr	sa	1.810	248.5	49.4	5.2	44.3	31.4	17.2	1.9	Very gravelly sandy loam	35.8	10.9	4.7
H037	St. Corona am Wechsel, AT	Gr	sa	0.760	247.5	49.2	0.0	37.3	34.9	26.9	0.9	Very gravelly sandy loam	42.5	14.7	6.5
H038	St. Corona am Wechsel, AT	Gr	sa	2.133	207.4	40.6	4.3	48.4	29.0	16.5	1.8	Very gravelly sandy loam	35.0	10.0	4.0
H126	Wimpassing an der Leitha, AT	Si	sa	0.038	14.2	2.2	0.0	5.2	26.5	62.0	6.3	Silt loam	41.7	52.1	6.1
H181	Ybbs, AT	Gr	sa	3.121	13.6	2.1	0.0	62.6	33.1	4.3	0.0	Extremely gravelly sand	35.9	2.1	0.2
H183	Ybbs, AT	Gr	sa	3.740	12.1	1.9	0.0	67.7	27.3	4.9	0.1	Extremely gravelly sand	31.5	1.5	0.1
H366	Steinakirchen, AT	Gr	sa	4.141	34.6	5.9	0.0	67.2	23.3	8.5	1.0	Extremely gravelly sand	31.3	3.9	0.7
H367	Steinakirchen, AT	Gr	sa	4.572	27.4	4.6	0.0	69.6	22.9	6.7	0.8	Extremely gravelly sand	29.7	3.1	0.4
H368	Steinakirchen, AT	Gr	sa	5.903	14.1	2.2	0.0	77.2	17.8	4.4	0.6	Extremely gravelly sand	23.5	1.3	0.1
H371	Steinakirchen, AT	Gr	sa	3.965	32.7	5.5	0.0	67.0	24.1	7.8	1.1	Extremely gravelly sand	32.0	3.9	0.6
H372	Steinakirchen, AT	Gr	sa	4.174	26.1	4.3	0.0	68.9	22.4	7.5	1.2	Extremely gravelly sand	31.0	3.2	0.4
H374	Steinakirchen, AT	Gr	sa	5.495	50.0	8.7	7.4	63.2	20.1	8.2	1.1	Extremely gravelly loamy sand	27.7	4.2	0.9
H405	Hagsdorf, AT	Si	sa	0.022	36.9	6.3	0.0	1.5	22.8	57.9	17.8	Silt loam	32.4	52.5	15.1
H493	Gajuri, NP	Si	sa	0.057	5.0	0.7	0.0	0.8	47.3	48.7	3.2	Sandy loam	56.0	43.2	0.8
H516	Omding, AT	Sa	gr	1.775	7.1	1.1	0.0	47.1	52.1	0.8	0.0	Very gravelly sand	53.3	1.3	0.0
H549	Gottsdorf, AT	Si	sa	0.033	13.0	2.0	0.0	0.2	30.0	64.9	4.9	Silt loam	37.4	56.3	6.3
H615	Pöchlarn, AT	Gr	sa	5.253	11.5	1.8	0.0	76.1	19.0	4.9	0.0	Extremely gravelly sand	24.4	1.1	0.1
H616	Simbach, AT	Sa	-	0.219	1.9	0.3	0.0	1.7	95.5	2.8	0.0	Sand	99.5	0.5	0.0
H617	Simbach, AT	Sa	si	0.105	9.0	1.4	0.0	5.2	60.1	33.7	1.0	Loamy sand	71.6	27.0	1.4
H637	Krems, AT	Gr	sa	2.336	151.2	28.9	0.0	53.1	32.1	12.8	2.0	Very gravelly loamy sand	35.3	9.1	3.2
H666	Heiligenkreuz, AT	Sa	si	0.072	4.3	0.6	0.0	0.2	53.0	45.4	1.4	Sandy loam	67.3	32.4	0.3
H668	Heiligenkreuz, AT	Si	sa	0.031	11.0	1.7	0.0	3.0	23.4	67.0	6.6	Silt loam	34.7	59.6	5.7
H693	Hollabrun, AT	Si	cl	0.005	72.6	13.1	0.0	0.0	8.4	56.4	35.2	Silty clay loam	10.5	53.7	35.8

**Fig. 2:**
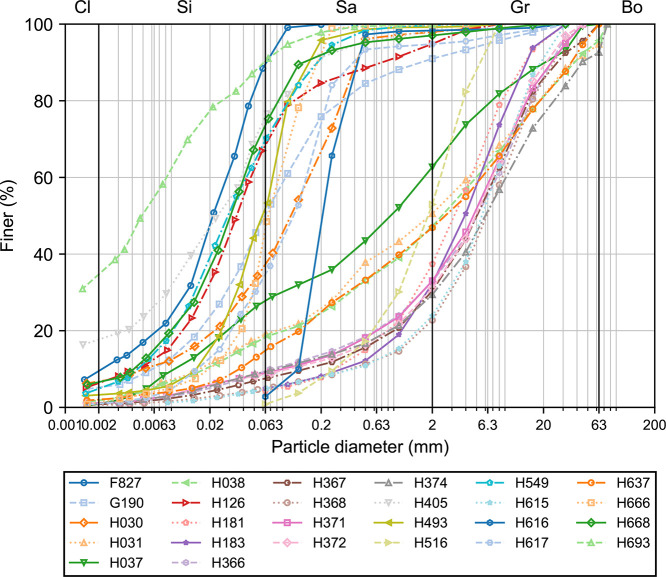
Particle size distributions of 26 different soil samples. The x-axis represents the particle diameter on a logarithmic scale, while the y-axis shows the percentage of finer particles. Each line corresponds to a different soil sample, distinguished by varying colors, linestyles and markers. The legend below the plot identifies each soil sample by its respective label.

The dataset contains a wide range of soil samples with varying particle sizes and compositions (Fig. 2). The average median particle diameter ($d_{50}$) is approximately 1.921 mm, indicating that, on average, the particles in the samples are relatively small. The average uniformity coefficient ($C_{U}$) is 50.250, suggesting that the soil samples have a wide range of particle sizes, indicating well graded soils. The average coefficient of curvature ($C_{C}$) is 9.362. The average percentages of cobbles (Co), gravel (Gr), sand (Sa), silt (Si) and clay (Cl) are 0.7 %, 33.8 %, 33.4 %, 27.9 % and 4.2 %, respectively, indicating that gravel and sand are significant components, while cobbles and clay are minor components of the soil samples.

The standard deviation for $d_{50}$ is 2.085 mm, indicating a wide range of median particle diameters among the samples. The standard deviation for $C_{U}$ is 74.452, suggesting very high variability in the uniformity coefficient, which further supports the presence of well graded soils. The standard deviation for $C_{C}$ is 14.861, indicating high variability in the coefficient of curvature among the samples. The standard deviations for Co, Gr, Sa, Si and Cl are 1.9 %, 31.0 %, 18.8 %, 25.0 % and 7.5 %, respectively, showing low to moderate variability in these components.

The median $d_{50}$ is 1.268 mm, which is lower than the mean, indicating a right-skewed distribution. The median $C_{U}$ is 16.500, significantly lower than the mean, suggesting a few samples with very high values. The median $C_{C}$ is 2.600, within the typical range for well-graded soils. The median percentages of Co, Gr, Sa, Si and Cl are 0.000, 0.408 (40.8 %), 0.282 (28.2 %), 0.169 (16.9 %) and 0.013 (1.3 %), respectively, indicating that more than half of the samples have no cobbles and the distributions of gravel, sand, silt and clay are skewed. The variables $d_{50}$, $C_{U}$, $C_{C}$, Co, Sa, Si and Cl are positively skewed (right-skewed), while the variable Gr is negatively skewed (left-skewed).

The smallest $d_{50}$ is 0.005 mm, indicating the presence of very fine particles in some samples. The smallest $C_{U}$ is 1.900, indicating that some samples are well-graded. The smallest $C_{C}$ is 0.300, below the typical range for well-graded soils. The smallest percentages of Co, Gr, Sa, Si and Cl are 0 %, 0 %, 8.4 %, 0.8 % and 0 %, respectively, indicating that some samples have no cobbles, gravel or clay and very little sand and silt.

The largest $d_{50}$ is 5.903 mm, indicating the presence of relatively large particles in some samples. The largest $C_{U}$ is 248.500, indicating extremely well graded soils in some samples. The largest $C_{C}$ is 49.400, much higher than the typical range for well-graded soils. The largest percentages of Co, Gr, Sa, Si and Cl are 7.4 %, 77.2 %, 95.5 %, 80.1 % and 35.2 %, respectively, indicating that some samples are predominantly gravel, sand or silt with significant amounts of clay in some cases.

## 4 Experimental Design, Materials and Methods

### 4.1 Soil sampling

The soil samples were collected using various sampling strategies, primarily during dynamic probing, where sampling was conducted at specific depths. Additionally, samples were obtained from trial pits and delivered by external companies. The sampling depths varied depending on the method and site conditions. All samples were stored in sealed buckets after collection to prevent contamination. Prior to analysis, the samples were homogenized using a cement mixer to achieve a representative distribution of particle sizes.

### 4.2 Dark chamber

**Fig. 3:**
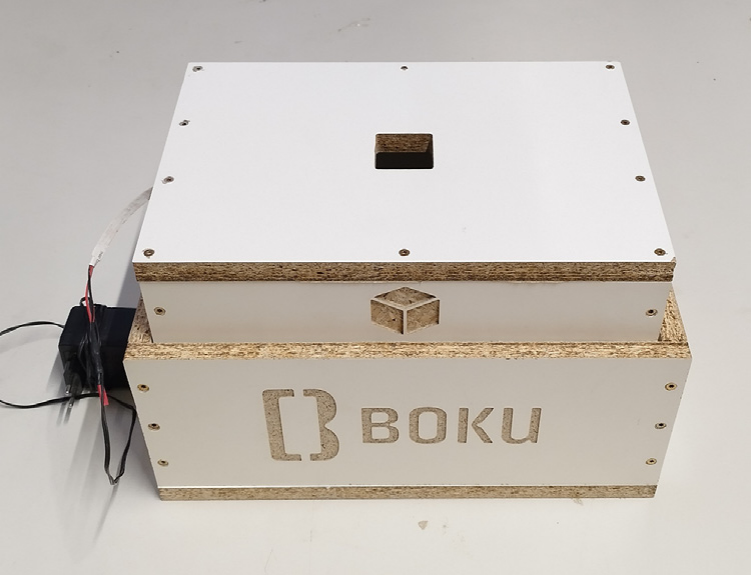
Experimental setup for capturing soil images. The smartphone is positioned on top of a dark chamber to minimize light reflection, with photos taken through a 45×45 mm opening. The distance from the soil surface to the camera lens is set to about 210 mm [2].

**Fig. 4:**
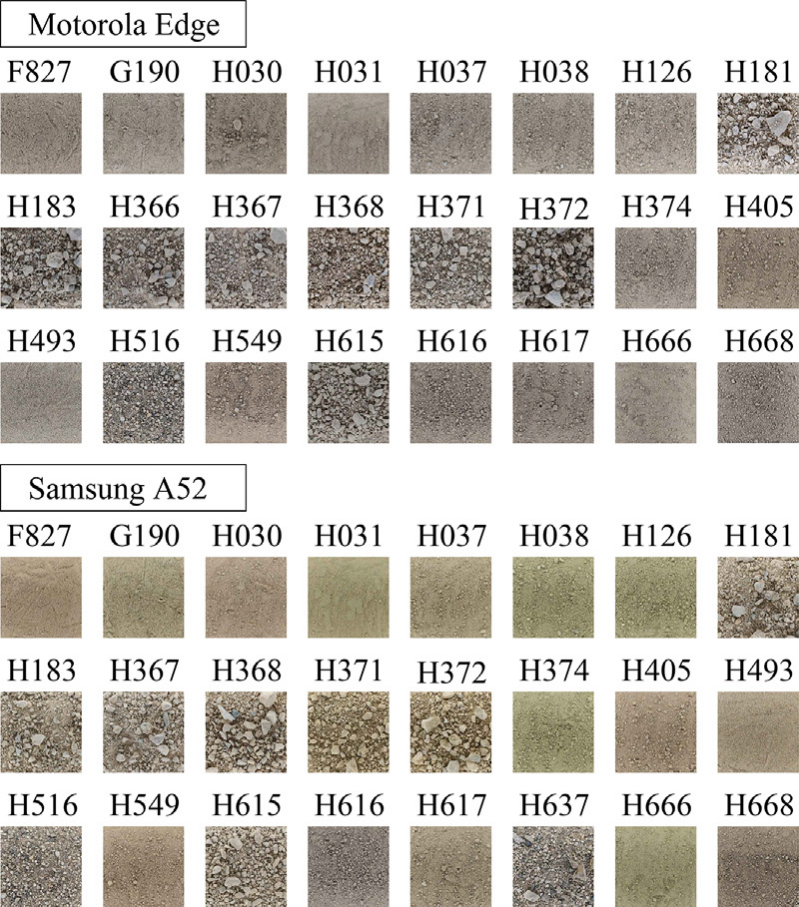
Sample images of soil F827 taken with Motorola Edge and Samsung A52 smartphones. The original images have resolutions of 4000×1800 and 6936×9248 pixels, respectively. Multiple images are taken for each soil sample to avoid biases, with the soil surface altered between shots [2].

Photographs are taken using smartphone cameras inside a dark chamber to prevent light reflection (Fig. 3). The smartphones are mounted at the top of the chamber and images are captured through a 45×45 mm aperture. The distance between the soil surface and the camera lenses is set to about 210 mm, as recommended by a previous study [10]. Since different smartphone cameras have varying angles-of-view, the photographed soil areas differ even when the distance remains constant. The inner dimensions of the chamber are designed to accommodate the areas captured by various smartphones from a 210 mm distance. The soil samples are homogenized using a cement mixer and then oven-dried at 105${}^{\circ }$C. The soil is dry-pluviated [11] from a height of 40 cm. Images are taken with Motorola Edge and Samsung A52 smartphones, with resolutions of 4000×1800 and 6936×9248 pixels, respectively (Table 2). Fig. 4 shows one (downscaled) image from each soil sample. To minimize bias, multiple images of the same soil are taken, with the soil surface being altered using various tools between shots. In total, 136 images are captured.

**Table 2:** Soil areas and pixel densities (PPM = pixel per mm) of the pictures taken with two smartphone cameras from the 210 mm distance to the soil surface.

Smartphone	Width	Length	PPM
	(pixels)	(pixels)	(pixels×mm)
Motorola Edge	1800	4000	11.5
Samsung A52	6936	9248	26.1

### 4.3 Particle size distribution

The PSD is determined by subdividing soil into discrete particle size classes using sieving and/or sedimentation methods [14]. For mixed soils, both sieving and sedimentation are performed. If 90 % or more of the particles are larger than 0.063 mm, sieving is used. If more than 10 % of the particles are smaller than 0.063 mm, sedimentation is used. For a complete grain size distribution curve, both methods are required.

#### 4.3.1 Sieving

**Fig. 5:**
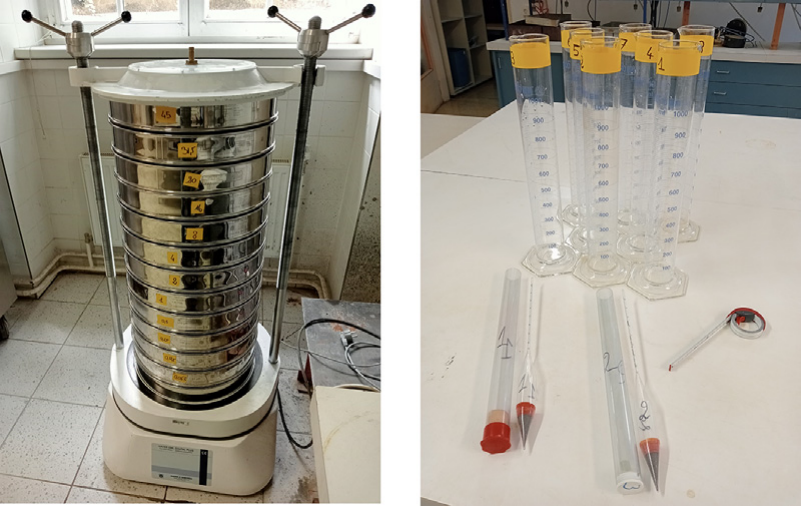
Mechanical sieve and hydrometer setups for soil particle size classification. Sieves compliant with ISO 565 [12] and ISO 3310 [13] standards classify soil samples, which are oven-dried and prepared as needed. For sedimentation analysis, non-dried specimens are dispersed in a liquid and measured with a calibrated hydrometer. Key parameters and temperature are recorded throughout the process.

Sieving separates soil into particle size classes using test sieves (Fig. 5(a)). Test sieves comply with ISO 565 [12] and ISO 3310 [13] standards and a range of sieves with apertures from 125 mm to 0.063 mm is used. Oven-drying is done at 105${}^{\circ }$C ± 5${}^{\circ }$C, unless the soil is susceptible to heating, in which case it is dried at 50${}^{\circ }$C. Wet preparation is used for soils with more than 10 % fines. A representative specimen is prepared by riffling or quartering and oven-drying. Minimum masses for dried specimens are specified based on particle diameter [14].

The fraction of soil passing each sieve is calculated using the equation:

(1) $$f_{n}=\frac{100(m_{1}+m_{2}+\ldots +m_{n})}{m}$$

where:

• $f_{n}$ is the fraction passing the sieve n (%)
• $m_{1}$ is the mass of soil passing the smallest mesh size (g)
• $m_{2},\ldots ,m_{n}$ are the masses of soil passing the consecutive sieves (g)
• m is the total dry mass of the specimen (g)

If the soil has been pretreated, the dry mass after pretreatment replaces m in the equation. Particles larger than 2 mm are excluded if insignificant and this should be reported as a percentage of the total mass.

#### 4.3.2 Sedimentation

Sedimentation separates particles based on their settling rates in a liquid. For sedimentation, a non-dried soil specimen is used and the water content and density of solid particles are determined separately (Fig. 5(b)). The soil is dispersed in a liquid using a mechanical shaker or stirrer and the suspension is transferred to a measuring cylinder for sedimentation analysis. Sedimentation is performed using a torpedo-shaped hydrometer, made of defect-free glass and calibrated for accuracy. Hydrometer readings are taken at specified intervals and the temperature of the suspension is recorded.

The total dry mass of the specimen is calculated using:

(2) $$m=\frac{m_{w}\cdot 100}{100+w}$$

where:

• m is the total dry mass (g)
• $m_{w}$ is the wet mass of soil (g)
• w is the water content (%)

The true hydrometer reading is calculated using:

(3) $$R_{h}=R_{h}^{\prime }+C_{m}$$

where:

• $R_{h}$ is the true hydrometer reading
• $R_{h}^{\prime }$ is the observed hydrometer reading
• $C_{m}$ is the meniscus correction

The effective depth $H_{r}$ is calculated from the true hydrometer reading using the hydrometer scale calibration. The equivalent particle diameter is calculated using Stokes law:

(4) $$d_{i}=\frac{0.005531\cdot H_{r}\left(\rho_{s}-1\right)}{\eta t}$$

where:

• $d_{i}$ is the equivalent particle diameter (mm)
• η is the dynamic viscosity of water
• $H_{r}$ is the effective depth of the hydrometer (mm)
• $\rho_{s}$ is the particle density (Mg/m${}^{3}$)
• t is the time (s)

The modified hydrometer reading is calculated using:

(5) $$R_{d}=R_{h}^{\prime }-R_{0}^{\prime }$$

where:

• $R_{d}$ is the modified hydrometer reading
• $R_{h}^{\prime }$ is the observed hydrometer reading
• $R_{0}^{\prime }$ is the observed hydrometer reading in the reference solution

The fraction smaller than the equivalent diameter is calculated using:

(6) $$K=\frac{100R_{d}\rho_{s}}{m}$$

where:

• K is the fraction smaller than the equivalent diameter (%)
• $\rho_{s}$ is the particle density (Mg/m${}^{3}$)
• m is the dry mass of the specimen (g)
• $R_{d}$ is the modified hydrometer reading

### 4.4 Machine learning approaches

Soil image datasets can be used to train machine learning models to predict particle size distributions [15], [16]. Buscombe, 2020 [15] developed a configurable machine-learning framework to estimate various sedimentological properties from photographic imagery. Tested on a dataset of 409 images of coarse soils, the model estimated grain size percentiles and equivalent sieve diameters directly from image features without requiring image scaling. The ground truth consisted of grain size distributions obtained following the on-screen manual method.

Lang et al., 2021 [16] introduced a CNN designed for grain size analysis of river systems using unmannend Aerial vehicle images. Their method involved training the CNN on 1491 coarse soil samples whereby the labelling was obtained from traditional image processing. They achieved a mean absolute error (MAE) of 1.1 cm for mean diameters.

In contrast, the approach of Soranzo et al., 2025 [2] focuses on both coarse and fine soils and derives the ground truth from standardised geotechnical testing [14]. Also, the PSD is described by means of curve fitting parameters rather than through characteristic particle sizes.

## Limitations

The dataset has the following limitations:

1. **Camera variability**: The images were taken using two different smartphone cameras (Motorola Edge and Samsung A52), which have different resolutions and angles-of-view. This variability may introduce inconsistencies in the image quality and the area of soil captured.
2. **Lighting conditions**: Although the images were captured in a dark chamber to minimize light reflection, any slight variations in lighting conditions could affect the consistency of the images.
3. **Soil surface alteration**: The soil surface was altered between shots to avoid biases. While this approach helps in reducing bias, it may also introduce variability in the soil surface conditions, which could affect the analysis.
4. **Sample preparation**: The soil samples were homogenized using a cement mixer and oven-dried. Any inconsistencies in the homogenization or drying process could affect the particle size distribution results.
5. **Resolution and scale**: The images have high resolutions (4000×1800 and 6936×9248 pixels), but the scale of the images may vary depending on the distance and angle of the camera. This could affect the accuracy of particle size predictions.
6. **Limited soil types**: The dataset includes a diverse range of soil samples, but it does not cover all possible soil types and conditions. This could limit the generalizability of the machine learning models trained on this dataset.
7. **Data size**: The dataset comprises 136 images and 26 CSV files. A larger dataset could provide more comprehensive training and validation of machine learning models. Partnering with an industrial collaborator, the dataset is already being expanded and the next machine learning model with the newly acquired data is under training. Concurrently, sensitivity analyses using artificial particle size distributions are being carried out to further enhance data diversity and robustness.

## Ethics Statement

The author has read and follow the ethical requirements for publication in Data in Brief and confirms that the current work does not involve human subjects, animal experiments or any data collected from social media platforms.

## Declaration of generative AI and AI-assisted technologies in the writing process

During the preparation of this work the author used ChatGPT in order to improve readability and language of the work under human oversight and control and after careful review and edit of the result to avoid authoritative-sounding output that can be incorrect, incomplete or biased. After using this tool/service, the author(s) reviewed and edited the content as needed and take(s) full responsibility for the content of the published article.

## CRediT authorship contribution statement

**E. Soranzo:** Conceptualization, Data curation, Formal analysis, Funding acquisition, Methodology, Project administration, Software, Supervision, Validation, Visualization, Writing – original draft, Writing – review & editing.

## Data Availability

ZenodoClose range images of soils and their particle size distributions (Original data).
